# Public Health Emergency Risk Communication and Social Media Reactions to an Errant Warning of a Ballistic Missile Threat — Hawaii, January 2018

**DOI:** 10.15585/mmwr.mm6807a2

**Published:** 2019-02-22

**Authors:** Bhavini Patel Murthy, Nevin Krishna, Terrance Jones, Amy Wolkin, Rachel Nonkin Avchen, Sara J. Vagi

**Affiliations:** ^1^Epidemic Intelligence Service, CDC; ^2^Division of State and Local Readiness, Center for Preparedness and Response, CDC; ^3^Office of Science and Public Health Practice, Center for Preparedness and Response, CDC.

On January 13, 2018, at 8:07 a.m. Hawaii Standard Time, an errant emergency alert was sent to persons in Hawaii. An employee at the Hawaii Emergency Management Agency (EMA) sent the errant alert via the Wireless Emergency Alert (WEA) system and the Emergency Alert System (EAS) during a ballistic missile preparedness drill, advising persons to seek shelter from an incoming ballistic missile. WEA delivers location-based warnings to wireless carrier systems, and EAS sends alerts via television and radio (*1*). After 38 minutes, at 8:45 a.m., Hawaii EMA retracted the alert via WEA and EAS (*2*). To understand the impact of the alert, social media responses to the errant message were analyzed. Data were extracted from Twitter* using a Boolean search for tweets (Twitter postings) posted on January 13 regarding the false alert. Tweets were analyzed during two 38-minute periods: 1) early (8:07–8:45 a.m.), the elapsed time the errant alert circulated until the correction was issued and 2) late (8:46–9:24 a.m.), the same amount of elapsed time after issuance of the correction. A total of 5,880 tweets during the early period and 8,650 tweets during the late period met the search criteria. Four themes emerged during the early period: information processing, information sharing, authentication, and emotional reaction. During the late period, information sharing and emotional reaction themes persisted; denunciation, insufficient knowledge to act, and mistrust of authority also emerged as themes. Understanding public interpretation, sharing, and reaction to social media messages related to emergencies can inform development and dissemination of accurate public health messages to save lives during a crisis.

The rapid dissemination of public health messaging is a component of information management, one of the six core domains of public health preparedness (*3*). The information management domain addresses public health communication and includes two capabilities: 1) emergency public information and warning and 2) information sharing. Emergency public information and warning is an essential capability for state and local public health preparedness and consists of the ability to develop, coordinate, and disseminate information, alerts, warnings, and notifications to the public and incident management responders. The information sharing capability consists of the ability to conduct multijurisdictional, multidisciplinary exchange of health-related information and situational awareness data among all levels of the government and the private sector (*3*).

Using Sysomos^†^ (version 1.47; Meltwater), an analysis of social media data from Twitter was performed by conducting a Boolean search to identify relevant tweets. The search used the terms “missile and Hawaii,” “ballistic,” “shelter,” “drill,” “threat,” “alert,” or “alarm” to identify tweets posted on the morning of January 13, 2018. Twitter data were used for this analysis because they are available in the public domain and easily accessible. Retweets (reposting the same tweet) and quote tweets (reposting the tweet with a comment at the top of the tweet) were excluded to limit the analysis to initial tweets.

All tweets were stratified into one of two periods. The early period consisted of tweets sent during the initial 38 minutes (8:07–8:45 a.m.), and the late period consisted of those sent in the 38 minutes after the false alarm retraction message was issued via EAS and WEA at 8:45 a.m. Tweets were coded using grounded theory, which is a systematic approach to analyze qualitative data and develop theories from those data (*4*). Themes were identified until theoretical saturation was reached. Atlas.ti^§^ software (version 8; Atlas.ti Scientific Software Development) was used for all exploratory qualitative analysis.

A total of 127,125 tweets were identified; after excluding 69,151 (54%) retweets and 43,444 (34%) quote tweets, 14,530 (11%) initial tweets remained for analysis. Among these, 5,880 (40%) were sent during the early period, and 8,650 (60%) were sent during the late period.

Four themes emerged from the Twitter data during the early period: 1) information processing; 2) information sharing; 3) authentication; and 4) emotional reaction. Information processing was defined as any indication of initial mental processing of the alert. Many information processing tweets dealt with coming to terms with the imminent missile threat (Table). Information sharing consisted of any attempt to disseminate the alert, often directed at other persons’ Twitter handles (user names). One Twitter user shared a tweet with the U.S. Indo-Pacific Command, the Defense Intelligence Agency, and the White House National Security Council. Authentication involved any attempt to validate the alert. Finally, emotional reaction was the expression of shock, fear, panic, or terror.

**TABLE:** Selected Twitter posts, by theme from the early* and late^†^ periods in response to an errant warning of a ballistic missile threat — Hawaii, January 13, 2018

Period/Theme	Description	Examples
**Early period**		
Information processing	Indication of mental processing of the alert	“Sirens going off in Hawaii, ballistic missile threat issued. What’s happening?”
		“Idk what’s going on.. but there’s a warning for a ballistic missile coming to Hawaii? [expletive deleted]”
Information sharing	Disseminating alert to others	“Just got an iPhone alert of inbound balistic [*sic*] missile in Hawaii. Said Not a Drill. @PacificCommand @DefenseIntel @WHNSC”
		“@ananavarro @TheRickWilson @AC360 Hawaii we all got emergency sirens on our phones ballistic missile inbound to Hawaii”
Authentication	Validating the alert	“Is this missile threat real?”
		“Where is news about the ballistic missile inbound to Hawaii?”
Emotional reaction	Expressing shock, fear, panic, or terror	“there’s a missile threat here right now guys. I love you all and I’m scared as [expletive deleted]”
		“Woke up and started crying after seeing the Hawaii missile alert. Called my parents and balled [*sic*] my eyes out because I was so worried.”
**Late period**		
Denunciation	Blaming the emergency warning and response	“How do you “accidentally” send out a whole [expletive deleted] emergency alert that says there's a missile coming to Hawaii and to take cover. AND TAKE THIRTY MINUTES TO CORRECT?!?”
		“To the person in #Hawaii who sent out that false alarm alert message about missile attack TO EVERY [expletive deleted] CELL PHONE...MOVE TO ANTARCTICA NOW! [emojis deleted] #that[expletive deleted]scaredeveryone @Hawaii_EMA”
Insufficient knowledge to act	Expressing lack of a response plan	“my friend & i were running around the hotel room freaking out because HOW DO WE TAKE SHELTER FROM A [expletive deleted] MISSILE?!”
		“Can you imagine waking up to an alert that says. “Take shelter there is a missile on the way” like Bruh. What shelter is there for a missile? That [expletive deleted] might as well say. “Aye Bruh. Missile on the way. Good luck”
Mistrust of authority	Doubting the emergency alert system and/or governmental response	“And now, should there be another ballistic missile threat, how can we trust it knowing the last one was a grave mistake???”
		“@Hawaii_EMA We all need to know who is behind this!!! . This is not a joke. . How can we trust the emergency alarm now? #hawaii #missile”

* 8:07–8:45 a.m. Hawaii Standard Time.

^†^ 8:46–9:24 a.m. Hawaii Standard Time (additional themes identified in addition to those in the early period).

During the late period, the information sharing and emotional reaction themes persisted, and three new themes emerged: 1) insufficient knowledge to act; 2) denunciation; and 3) mistrust of authority (Table). These new themes are fundamentally different from those expressed during the early period and reflect reactions and responses to misinformation. Insufficient knowledge to act involved reacting to the lack of a response plan, particularly not knowing how to properly take shelter. Denunciation consisted of blaming the emergency warning and response, particularly criticizing the time it took to correct the alert. Finally, mistrust of authority involved doubting the emergency alert system or governmental response.

## Discussion

Public health messaging during an emergency is complicated by how messages are perceived and interpreted by different persons. Emergency messages need to be coordinated across multiple platforms to ensure that accurate and timely information is appropriately disseminated to the target audience (*5*). Social media can be an effective tool to manage public health messaging during an emergency, and social media reactions to a perceived threat highlight the complexity of sharing critical information.

Reactions on social media reveal that some social media users lacked awareness about actions to take when faced with a nuclear threat. CDC developed guidance describing what persons in an affected area should do in response to a number of different types of emergencies, including a ballistic missile strike (*6*).

The Hawaii EMA’s investigation of the errant emergency alert identified multiple contributing factors that led to the false alarm (*7*). The alarm notification occurred during a shift change, and there was a lack of understanding that the notification was meant to be a drill. The Federal Communications Commission (FCC) report noted that “the employee who triggered the false alert believed that the missile threat was real… In other words, the employee intended to initiate a real-world emergency alert based on that employee’s mistaken belief that Hawaii had come under a ballistic missile attack. This fundamental misunderstanding played a critical role in the initiation of the false alert.” (*8*). Furthermore, the exercise plans did not document a process for disseminating an all clear message (*7*).

As the situation unfolded, several public authorities posted information on Twitter stating that the alert was a false alarm. However, according to the FCC report, the established ballistic missile alert checklist did not include a step to notify the Hawaii EMA’s public information officer responsible for communicating information to the public, media, other agencies, and other stakeholders during an incident. Finally, the FCC report noted that there was no credentialed two-person requirement to separately log in and approve the transmission of a ballistic missile alert message (*8*).

Missile alert exercises have not been conducted regularly in decades (*9*). A large proportion of the U.S. population alive today did not experience, or are too young to recall, exercises conducted to defend against threats faced by the United States during the Cold War era. The lack of a collective memory of missile alert drills coupled with the present-day ability to instantaneously share information through social media can affect societal reactions. To improve risk communication, additional research is needed to understand human reactions to emergencies in the social media age so that timely public health messages can be developed and disseminated to save lives.

The findings in this report are subject to at least two limitations. First, because the qualitative coding could be subjective, the sincerity and tone of the tweets could have been misinterpreted. Second, although quote tweets add to the original content through framing, labeling, magnifying, and elaborating on an initial tweet, they were excluded from this analysis to focus on the initial tweets. It is likely, however, that themes in quote tweets are similar to those in initial tweets.

Public information officers, communication specialists, and others responsible for planning and creating urgent communications during an emergency incident should consider the behavioral themes identified in this report when creating messages for public dissemination. Social media provides public health authorities with the capability to convey timely messages, address societal reactions during each phase of a crisis, and establish credibility to avoid mistrust and denunciation of a public health message. Alerts should include clear instructions for persons in the affected area to carry out during an emergency.

**Summary**

**What is already known about this topic?**

Social media platforms are widely used to share information and disseminate alerts and warnings.

**What is added by this report?**

After an errant ballistic missile alert, social media reactions revealed how the public interprets, shares, and responds to information during an evolving threat. This knowledge can guide emergency risk communicators to develop timely and effective social media messages than can protect lives.

**What are the implications for public health practice?**

Social media can be an effective tool to send urgent messages during a public health emergency. Public health practitioners need to improve messaging during emergency risk communications to address the public’s needs during each phase of an unfolding crisis to protect and save lives.

## Footnotes

* Twitter is an online social networking service where users can exchange short messages, or tweets, with one another.

† https://sysomos.com/.

§ https://atlasti.com/.
